# Clinical and demographic features among patients with type 1 diabetes mellitus in Henan, China

**DOI:** 10.1186/s12902-021-00799-2

**Published:** 2021-06-28

**Authors:** Liguo Yang, Guangxing Yang, Xialian Li

**Affiliations:** 1 Department of Endocrinology, The First Affiliated Hospital of Zhengzhou University, 450052 Zhengzhou, Henan, China; 2 Department of Cardioangiology, The First Affiliated Hospital of Zhengzhou University, 450052 Zhengzhou, Henan, China

**Keywords:** Type 1 diabetes, Epidemiology, Chinese

## Abstract

**Background:**

The hallmark of type 1 diabetes (T1D) is an absolute lack of insulin. However, many studies showed a tendency to heterogeneity in TID. We aimed to investigate the demographic and clinical characteristics in T1D and the differences in young-onset and adult-onset patients.

**Methods:**

This retrospective study was conducted among 1943 patients with clinically diagnosed T1D. Medical records on patients’ demographics, anthropometric measurements, and clinical manifestation were collected. According to the age at onset, the newly diagnosed patients were divided into the young-onset group (< 18 years, 234 patients, mean age 11 years) and adult-onset group (≥ 18 years, 219 patients, mean age 27 years). Pancreatic β-cell function was assessed by fasting C-peptide (FCP) and 2-h C-peptide (2-h CP).

**Results:**

The median age of patients at disease onset was 22 years. The median duration of patients was 3 years. The overall median glycated hemoglobin (HbA1c) value was 10.3 % [89(mmol/mol)]. The prevalence of diabetic retinopathy was 25.1 %. The overall rate of DKA at onset in the new-onset patients was 59.6 %. The frequency of overall dyslipidemia was 37.8 %. The most frequent dyslipidemia was low high-density lipoprotein-cholesterol (HDL) (29 %). The proportion of patients with anti-glutamic acid decarboxylase (GADA), insulin antibody (IAA) and islet cell antibody (ICA) were 28.1 %, 6.4 % and 21.6 %, respectively. The mean HbA1c showed a downward trend with age. Increasing or decreasing trends of overweight and obesity in this population during the period 2012 to 2018 was not found.

Compared with young-onset T1D, adult-onset patients comprised better islet function (FCP: 0.4 vs. 0.3 ng/ml, *P* < 0.001; 2-h CP: 0.9 vs. 0.7 ng/ml *P* < 0.001, respectively) and glycemic control [12.9 % (117mmol/mol) vs. 11.7 % (104mmol/mol), *P* < 0.001], higher prevalence of diabetes condition in the male gender (64.4 % vs. 51.3 %, *P* = 0.006), higher proportion of obesity or overweight (24.6 % vs. 9.5 %, *P* = 0.002), higher frequency of GADA (33.7 % vs. 23.3 %, *P* = 0.025), and lower frequency of diabetic ketoacidosis at disease onset (64.5 % vs. 43.5 %, *P* < 0.001).

**Conclusions:**

This population was characterized by poor overall blood glucose control, high prevalence of DKA, dyslipidemia and diabetic retinopathy, and low prevalence of islet-related antibodies, and overweight or obesity. Adult-onset patients with T1D were not uncommon and had better clinical manifestations than young-onset patients. Any findings related to body mass index (BMI) and autoantibodies should be considered strictly exploratory due to excessive missing data.

## Background

Type 1 diabetes mellitus (T1D) is a chronic autoimmune disease in which endogenous insulin production is severely compromised by an immune-mediated injury of pancreatic β-cells[1]. The estimations of T1D incidence based on population-based registries showed a variation in the incidence all over the world during the past 2 decades. On the whole, the incidence around the world has been on the rise[2–5], but the incidence in some countries is decreasing, such as Finland[6]. The epidemiological data from China showed that the incidence of T1D has been on the rise[7–9]. George Eisenbarth put forward a conceptional model for the pathogenesis of T1D before 30 years. It held that both environmental risk and genetic susceptibility promote the development of T1D. However, the rise in incidence is too rapid to be explained by increased transmission of susceptibility haplotypes from one generation to the next. Furthermore, some studies manifested that as time goes on, high-risk HLA genotypes were becoming not so frequent as before in youth with T1D[10, 11]. Therefore, the increasing environmental pressure may play an increasingly important role in the occurring of T1D. There are many environmental factors, such as obesity, infant and adult diet, vitamin D deficiency, early exposure to islet immune-related viruses (such as enterovirus) and the decline of intestinal microbial diversity. These are still incompletely understood.

T1D is one of the most common chronic diseases of childhood, but it can occur at any age, even in the 8th and 9th decades of life[12]. American Diabetes Association (ADA) reported that the patients of adult-onset T1D was increasing and with up to 50 % of cases occurred in adulthood [13]. A study in China reported that 65.3 % cases of new-onset T1D occurred among adults[14]. Age of onset is a major driver of T1D heterogeneity [15]. Previous studies showed that patients presenting with T1D in adults were characterized by a longer symptomatic period before diagnosis, a better preserved β-cell function, and a reduced frequency of IAA, compared with young-onset subjects.

Considerable heterogeneity exists in clinical, immunologic and epidemiological characteristics of T1D based on ethnicity and country[15–17]. According to Multinational Project for Childhood Diabetes (DIAMOND), the Chinese is among populations with the lowest incidence of T1D [18], maybe resulting from the counterbalancing between protective DQB1 and susceptible DRB1[19]. However, in China, a region with large population base, a linear increase in diabetes incidence may lead to a substantial increase in the number of patients with T1D. It will eventually bring about a major increase in the estimated health expenditure which have a great economic impact on individuals and their families[20]. Therefore, it is of great value to investigate the situation of hospitalized patients for recognizing and addressing preventable medical problems. Consequently, we aim to investigate demographic, clinical characteristics of patients with T1D, as well as the pattern of being overweight or obesity changes in Henan in recent years.

## Materials and methods

### Study design

This was a hospital-based, retrospective study from the first affiliated hospital of Zhengzhou university that is a tertiary medical center in Zhengzhou city, Henan Province, China. Inpatients in this hospital included urban as well as rural population. The participants in our study covered both pediatric and adult patients with T1D who were diagnosed and treated by pediatricians or endocrinologists. Quite a few patients spent the critical period in the emergency department due to diabetic ketoacidosis before transferring to the general ward.

The medical records of T1D among all inpatients in our hospital from Jan 2012 to Dec 2018 were reviewed in hospital information system. All study patients had the diagnosis of T1D made by endocrinologists and documented in the hospital’s case database. We retrospectively evaluated all patients’ medical records and collected data on patients’ demographics, clinical manifestations, and metabolic indicators. Proteinuria and urine acetone bodies were assessed by presenting protein and (or) acetone bodies in urine routine examination (clean and interrupted morning urine was collected, and then was checked by an automatic analyzer). Retinopathy was defined by hemangioma, exudation, bleeding, or more serious proliferative lesions on one or both sides of the fundus from retinal photographs.

The study was carried out in compliance with the declaration of Helsinki. The study was approved by the Ethical Committee of the First Affiliated Hospital of Zhengzhou University (Number: 2020-KY-417). Because this was a retrospective observational study, and de-identified information was used for the analysis, informed consents were waived as part of the Institutional Review Board of the First Affiliated Hospital of Zhengzhou University approval.

### Study population

The inclusion criteria for T1D patients were determined by clinical characteristics. Based on the World Health Organization (WHO) reports for the classification of diabetes[21], the ADA descriptions of T1D[12], all participants should be dependent on insulin at disease onset, diagnosed with T1D by an endocrinologist, and met at least 1 of the followings: (1) classic symptoms of diabetes-related metabolic disorders at disease onset, (2) fasting C-peptide levels < 0.60ng/ml at initial diagnosis[22], (3) recurrent diabetic ketosis or ketoacidosis, (4) being positive for one or more of diabetes-related autoantibodies. If the diagnosis was uncertain, they will be further confirmed by expert committee for final judgement. Nine patients diagnosed with diabetes before age 6 months were excluded from the analysis. Fifty patients were excluded from the study because all laboratory test results could not be found in the inpatient system. Eventually 1943 T1D patients were enrolled in this study. The number of patients with different variables included in this study, may vary due to missing data.

In this study, patients aged 18 or above were defined as adults. Patients under 18 is defined as a child or adolescent. For 453 newly diagnosed patients with T1D, according to the age of onset, it was divided into the young-onset group (< 18 years, 234 patients) and adult-onset group (≥ 18 years, 219 patients).

### Measurement and Definition

Body mass index (BMI) was calculated using the measured weight (kg) divided by the square of measured height (m). Age-specific and sex-specific BMI criteria for overweight and obese categories for children and adolescents aged < 18 years were derived from the International Obesity Task Force (IOTF definition). For patients aged ≥ 18 years, overweight and obesity were categorized according to WHO criteria for Asian population as BMI ≥ 23 to < 27.5 kg/m^2^ and ≥ 27.5 kg/m^2^, respectively.

Chinese Guidelines on Prevention and Treatment of Dyslipidemia in Adults cut-offs points [total cholesterol (TC) ≥ 6.2mmol/l (240 mg/dl), triglyceride (TG) ≥ 2.3mmol/l (200 mg/dl), low-density lipoprotein cholesterol (LDL-C) ≥ 4.1mmol/l (160 mg/dl), high-density lipoprotein cholesterol (HDL-C) < 1.0mmol/l (40 mg/dl)]for adults and Experts Consensus for Prevention and Treatment of Dyslipidemia in Children and Adolescents cut-offs points [TC ≥ 5.2mmol/l (200 mg/dl), TG ≥ 1.7mmol/l (150 mg/dl), LDL-C ≥ 3.4mmol/l (130 mg/dl), HDL-C<1.0mmol/l (40 mg/dl )]for pediatric patients were adopted. Dyslipidemia was defined by the presence of one or more abnormal serum lipid concentration.

Diabetic ketoacidosis (DKA) was defined by plasma glucose ≥ 13.9 mmol/L (250 mg/dl), blood bicarbonate < 15 mmol/L and or PH < 7.3, and elevated level of ketones in urine or blood.

### Laboratory analysis

The levels of glutamic acid decarboxylase antibody (GADA), islet cell antibody (ICA), and insulin antibody (IAA) were tested using the radioligand assay (Amon Biotechnology Company, Germany). The inter- and intra-assay variation coefficients of the GADA assay were 7.1–10.8 % and 4.9–8.3 %, respectively. The sensitivity and specificity of the GADA assay were 82 and 98 %, respectively. The inter- and intra-assay variation coefficients of the IAA assay were 7.0 − 11.0 % and 5.8 − 8.3 %, respectively. The sensitivity and specificity of the IAA assay were 50 and 97 %, respectively. The inter- and intra-assay variation coefficients of the ICA assay were 4.2–9.8 % and 4.0-9.9 %, respectively. The sensitivity and specificity of the ICA assay were 92.6 %, 81.3 %, respectively.

C peptide (CP) was determined by chemiluminescence (ADVIA Centaur, Acta Siemens, Munich, Germany). The inter- and intra-assay coefficients of the variation were 3.7–4.1 % and 1.0–3.3 %, respectively. The normal range for C-peptide was 1.1–4.4 ng/ml.

The hemoglobin A1c (HbA1c) levels were measured by automated liquid chromatography (HLC723G8, Tosoh). The inter- and intra-assay variation coefficients were less than 3 and 1 %, respectively.

In our hospital, we generally perform these tests for patients who can or have been diagnosed with T1D. Pancreatic islet autoantibodies are usually checked with fasting blood on the morning of the second day of hospitalization. The C-peptide test is usually done together with OGTT, usually when the fasting blood glucose is adjusted to below 8 mmol/l after blood glucose management.

### Statistical analysis

Statistical analysis was performed with Statistical Product and Service Solutions (SPSS) version 24.0, Statistical analysis system (SAS) version 9.4 and GraphPad Prism 7. All *P* values of less than 0.05 were regarded as being statistically significant. Participants’ characteristics were defined using descriptive statistics. Continuous variables were presented as mean ± SD or median (25th, 75th quartile). The categorical variables were expressed as number of cases and percentage of patients affected n (%). Two independent sample t-test for normal distributed variables, Mann-Whitney *U* Test for nonparametric statistics and chi-square test for categorical variables were used to examine differences in demographic and clinical characteristics between young-onset and adult-onset groups. Chi-square trend test was analyzed in SAS to observe linear correlation between proportion of overweight or obesity and year. The chart of the smoothed HbA1c mean versus age has been made in GraphPad Prism 7. For variables with a small percentage of missing data (≤ 10 %), we adopted the available-case analysis. For variables with a large percentage of missing data (> 10 %), we adopted the multiple imputation methods.

## Results

### Clinical and demographic characteristics of all patients

This analysis consisted of 1943 subjects [793 children and adolescents (< 18 years) and 1150 adults (≥ 18 years)]with a mean age of 28 ± 15 years (range 6 months to 83 years), and a mean duration of 5 ± 5 years (range 0 day to 38 years, the duration of newly diagnosed patients were regarded as 0 day.). As shown in Table 1 and 50.6 % of cases were male. The median age of patients at disease onset was 22 years old. Among 1943 patients with T1D, HbA1c values were obtainable in 1725 patients. The overall median (interquartile range) HbA1c value was 10.3 (8.2,12.5) % or 89(66,113) mmol/mol. Height and weight data were obtainable in 1250 patients. Overall, 16.9 % of participants were overweight or obesity. Lipid values were obtainable in 1646 patients. The frequency of dyslipidemia was 37.8 %. The most frequent dyslipidemia was low high-density lipoprotein cholesterol (HDL) (29 %). We observed that 25.1 % of patients presented with retinopathy. The proportion of patients with GADA, IAA and ICA were 28.1 %, 6.4% and 21.6 %, respectively. Besides, 59.6 % of newly diagnosed patients occurred diabetic ketoacidosis (DKA) at onset.

**Table 1:** Clinical and demographic characteristics of all patients (*N* = 1943)

Characteristic	Missing (%)	n (%) or Median (IQR)
Male	0 (0)	984(50.6)
Current age(year)	0 (0)	26(16, 38)
Age at onset(year)	0 (0)	22(13, 32)
Duration(year)	453 (23.3)	3(1,8)
HbA1c (%)	218 (11.2)	10.3(8.2, 12.5)
HbA1c (mmol/mol)	218 (11.2)	89(66,113)
Overweight or Obesity	693 (35.7)	211(16.9)
Overweight	693 (35.7)	171(13.7)
Obesity	693 (35.7)	40(3.2)
Retinopathy	82(4.2)	468(25.1)
Glutamic acid decarboxylase antibody	772 (39.7)	329(28.1)
Islet-cell antibodies	779(40.1)	252(21.6)
Insulin antibodies	779 (40.1)	74(6.4)
Dyslipidemia	297(15.3)	623 (37.8)
High total cholesterol	301 (15.5)	140(8.5)
High triglyceride	305 (15.7)	185(11.3)
Low high-density lipoprotein cholesterol	312 (16.1)	472(29.0)
High low-density lipoprotein cholesterol	288 (14.8)	116(7.1)
DKA at onset in newly diagnosed patients	52(11.5)	239(59.6)
Fasting C-peptide (ng/ml)	214 (11.0)	0.3 (0.1, 0.5)
2-h C-peptide (ng/ml)	214 (11.0)	0.8 (0.3, 1.3)

Comment: Values were presented as numbers of patients(percentages) or median (interquartile range).

### The mean level of HbA1c in different age interval of T1D patients

**Fig. 1:**
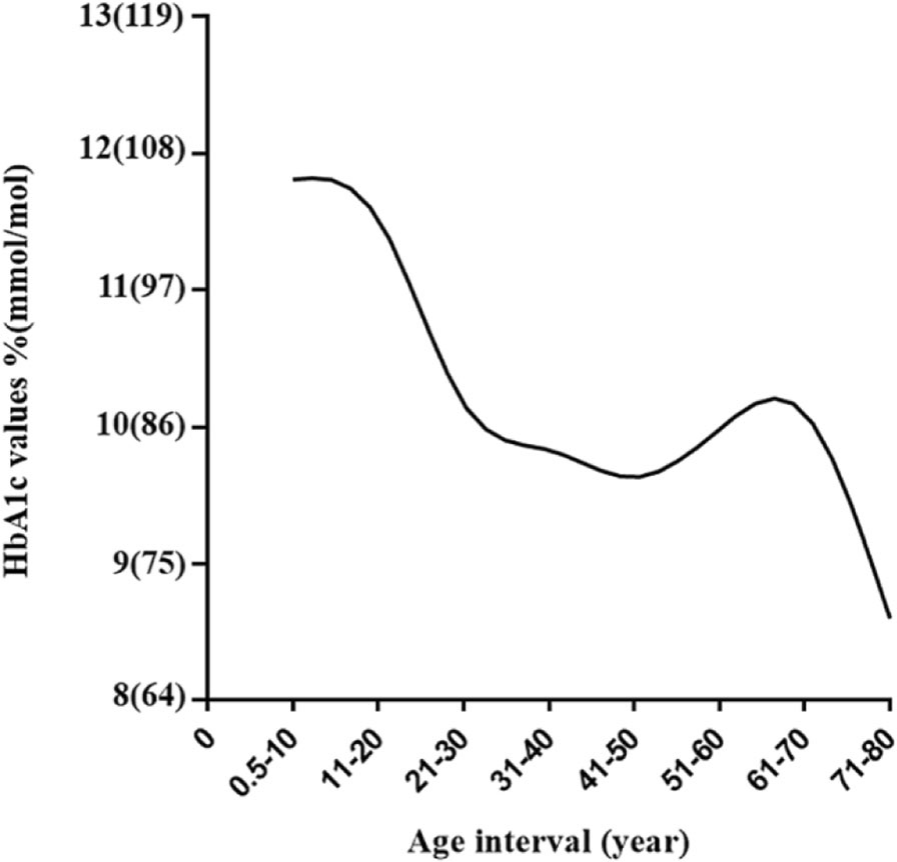
The mean levels of HbA1c in different age interval

From Fig. 1, on the whole, the mean HbA1c value showed a downward trend with age, and the peak was in the age interval under 10 years. Between 21 and 51 years, the mean HbA1c value seemed to be in a plateau period showing slow declining. There was also a second peak in the age interval 61–70 years.

### Temporal changes in proportion of overweight and obesity

**Fig. 2:**
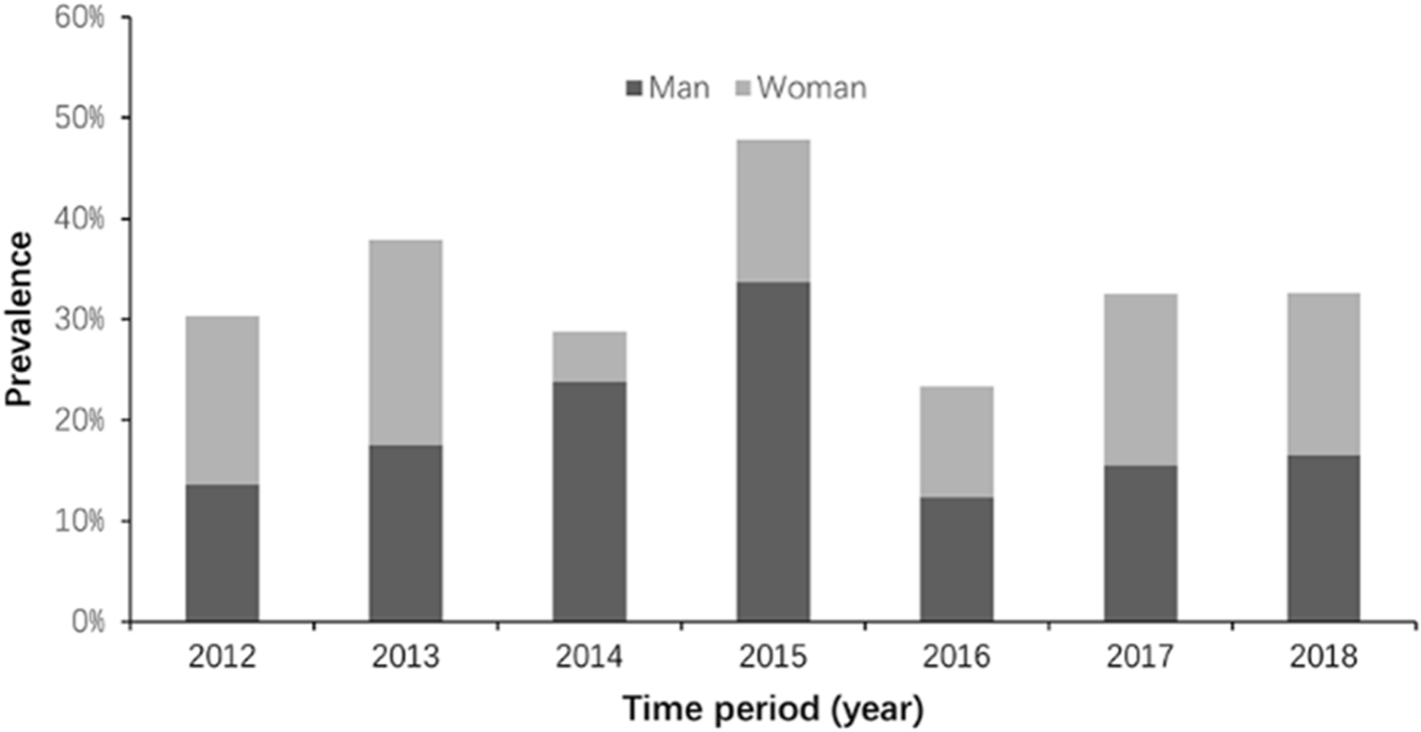
Trends in the prevalence of overweight and obesity among T1D patients by gender (2012–2018)

**Fig. 3:**
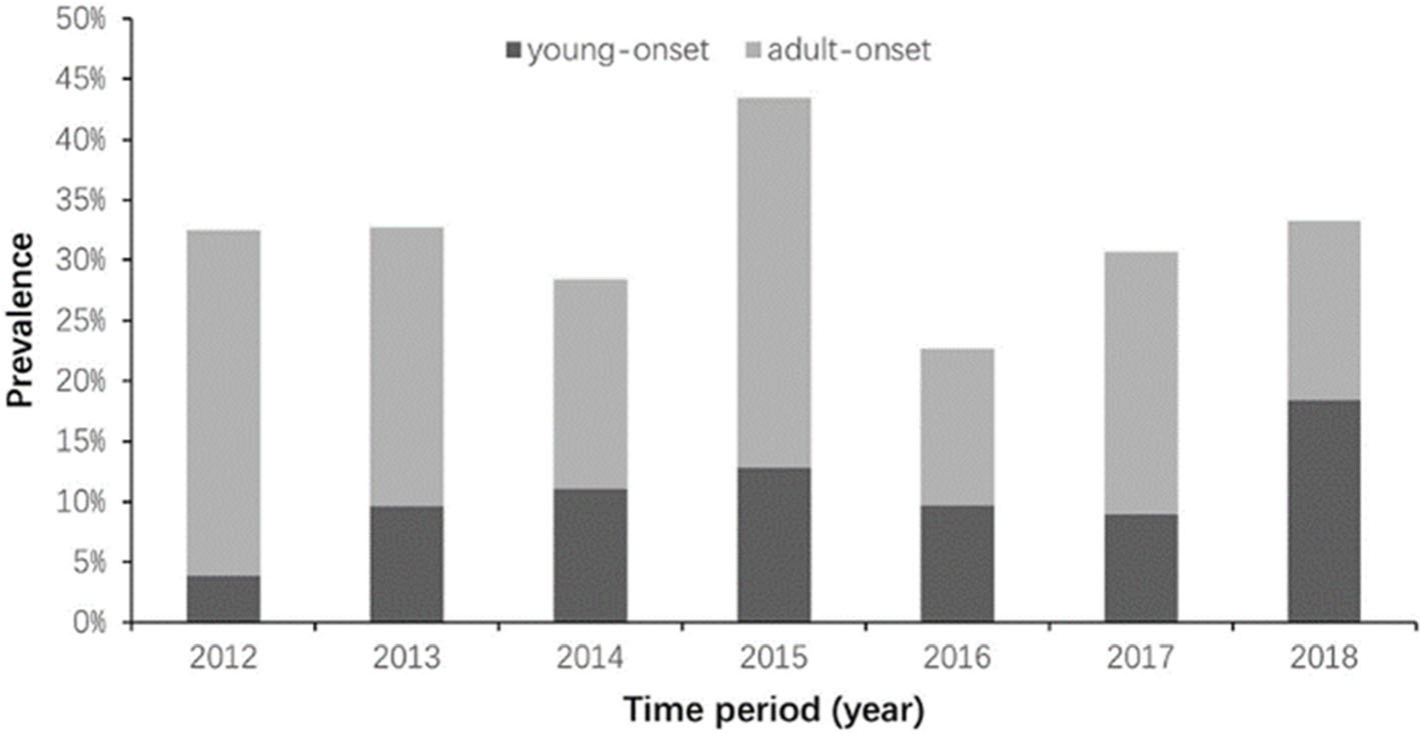
Trends in the prevalence of overweight and obesity among T1D patients by age at onset (2012–2018)

The overall prevalence of overweight or obesity during 2012 and 2018 was 3.3 %, 14.1 %,11.7 %, 20.2 %, 10.8 %, 16.0 and 23.9 %, separately. Chi-square trend test showed that there was no significantly linear correlation between overall prevalence of overweight or obesity and year (*Z* = 0.1501, *P* = 0.699). As shown in Figs. 2 and 3, the prevalence of overweight or obesity in the adult-onset group is higher than that in the young-onset group (all *P* < 0.05), and there is no difference between men and women (all *P* > 0.05).

### Comparison of characteristics in newly diagnosed young-onset and adult-onset patients

A total of 453 patients who were newly diagnosed in our hospital were selected to compare characteristics in young-onset and adult-onset group. As shown in Table 2, the prevalence of diabetes family histories in young-onset and adult-onset group were21.5 and 23.4 %, separately; but no significant difference was found in two groups. We observed that the adult-onset group had a higher proportion of overweight or obesity (24.6 % vs. 9.5 %, *P* = 0.002) than young-onset group. The young-onset participants had a higher median HbA1c value than the adult-onset patients [12.9 % (117mmol/mol) vs. 11.7 % (104mmol/mol)], *P* < 0.001). The frequency of diabetic ketoacidosis in young-onset group was significantly higher than that in adult-onset group (64.5 % vs. 43.5 %, *P* < 0.001). The adult-onset group had significantly higher FCP and 2-h CP values (0.4 vs. 0.3ng/ml, *P* < 0.001; 0.9 vs. 0.7 ng/ml *P* < 0.001, respectively), whereas lower proportion of undetectable/ detectable C-peptide values (3 % vs. 11 %, *P* = 0.003) than young-onset patients. The overall frequency of dyslipidemia was 54.7 % and was more in adult-onset group (58.2 % vs. 51.3 %, *P* = 0.212). The frequency of high level of TC in young-onset group was significantly higher than that in the other group (16.6 % vs. 8.8 %, *P* = 0.029). The prevalence of GADA in adult-onset group was higher than that in young-onset group (33.7 % vs. 23.3 %, *P* = 0.025).

**Table 2:** Comparison between new-diagnosed young-onset and adult-onset patients with T1D

	Young-onset (*n* = 234)	Adult-onset (*n* = 219)	*P*-value
Male (%)	120(51.3)	141(64.4)	0.006
Age at diagnosis(year)	11(7,13)	28(24,37)	< 0.001
HbA1c (%)	12.9 ± 2.4	11.7 ± 3.0	< 0.001
HbA1c(mmol/mol)	117 ± 3	104 ± 9	< 0.001
Diabetic ketoacidosis at onset	148(64.5)	91(43.5)	< 0.001
Overweight or Obesity	12(9.5)	32(24.6)	0.002
Overweight	6(4.8)	23(17.7)	0.001
Obesity	6(4.8)	9(6.9)	0.597
Glutamic acid decarboxylase antibody	49(23.3)	61(33.7)	0.025
Islet-cell antibodies	43(20.6)	46(25.4)	0.277
Insulin antibodies	7(3.3)	3(1.7)	0.351
Dyslipidemia	98(51.3)	106(58.2)	0.212
High total cholesterol	31(16.6)	16(8.8)	0.029
High triglyceride	36(18.8)	28(15.4)	0.412
Low high-density lipoprotein cholesterol	82(42.9)	92(51.4)	0.118
High low-density lipoprotein cholesterol	14(7.4)	17(9.5)	0.574
Urine acetone bodies	177(77.0)	151(71.9)	0.230
Proteinuria	32(14.0)	28(13.4)	0.890
Diabetes family history	50(21.5)	51(23.4)	0.652
Fasting plasma glucose (mmol/l)	6.6 (5.2,8.0)	7.3 (5.6,10.0)	0.051
Fasting C-peptide (ng/ml)	0.3 (0.2,0.5)	0.4 (0.2,0.7)	< 0.001
2-h C-peptide (ng/ml)	0.7 (0.4,1.1)	0. 9 (0.6,1.8)	< 0.001
Total cholesterol (mmol/l)	4.0 (3.4,4.9)	4.1 (3.3,4.8)	0.785
Triglyceride (mmol/l)	1.0 (0.7, 1.6)	1.2 (0.8,1.9)	0.347
High-density lipoprotein cholesterol (mmol/l)	1.1 (0.9,1.4)	1.0 (0.9,1.2)	0.006
Low-density lipoprotein cholesterol (mmol/l)	2.3 (1.9,2.9)	2.5(2.0,3.3)	0.057
Undetectable/ detectable C-peptide	5/179	19/166	0.003

Comments: Values were presented as numbers of patients(percentages), median (interquartile range), or means ± SD

## Discussion

In this study, we retrospectively studied demographic and clinical features of T1D. We found that the study patients were characterized by poor blood glucose control, high prevalence of acute and chronic complications (manifested by DKA, retinopathy, and dyslipidemia) and low detection rate of diabetes-related antibodies. On the whole, the mean HbA1c value showed a downward trend with age. There was no significantly linear correlation between overall prevalence of overweight or obesity and year from 2012 to 2018. Compared with young-onset T1D, adult-onset patients comprised higher proportion of diabetes condition in the male gender, obesity or overweight and positivity of GADA, and better clinical manifestations.

### Comparison of Demographic and Clinical Features between this Population and Others

In this analysis, we found that 59.6 % of newly diagnosed patients occurred diabetic ketoacidosis (DKA) at onset. This was higher than that from another study in China (50.1 %)[23]. In terms of the methodological discrepancies between studies, our studies revealed a high prevalence of DKA, which may be related to poorer metabolic control. This study showed glycemic control was poor among patients with T1D, in whom the median level of HbA1c was 10.3 % (89 mmol/mol). It was similar to the published data from another cross-sectional study in China[24], which showed that mean HbA1c value was > 75 mmol/mol (9.0 %). We also found that the mean HbA1c values were in a high level in all age interval and showed a downward trend with age (Fig. 1). A study[25] in the United States showed that the mean HbA1c values was 8.4 % (68 mmol/mol), and only a minority of patients with T1D achieved ADA goals for HbA1c. The differences of level of HbA1c values between different studies may be due to ethnicity, clinical characteristics, parental education, and income level, but the most important factor could be overall quality of care. Although all T1D patients in this study received insulin therapy, the specific form of insulin therapy is unclear (most T1D patients in our hospital are treated with basal-bolus therapy, a minority of patients are treated with insulin pump therapy). Overall, the high level of HbA1c values in this study mean that patients had higher risk of onset and progression of microvascular complications of T1D[26, 27]. The result showed that the overall prevalence of retinopathy in our data was 25.2 %, which was higher than that reported in Swedish(21 %) [28], Norway (16 %)[29], USA (14 %) [30]and Germany (11 %) [31],regardless of shorter diabetic duration in this study. This may be resulted from poor glucose control, earlier and more frequent screening in our hospital, or clinical heterogeneity. Overt complications (proliferative retinopathy) remain rare in the adolescent population with T1D. Early indicators and pathological changes are clinically detectable in adolescents after 2- to 5-year disease duration[32, 33].

Weight change in TID is complex. Factors that may affect weight gain in patients with T1DM include the level of glycemic control, intensive insulin treatment, pattern of treatment, pubertal status, the presence of eating disorders and appearance of complications (such as thyroid disease or gastric disease)[34]. There are many studies that reported the prevalence of overweight and obesity of T1D, which varies from 21.0 to 53.8 % in different countries with different criteria of overweight or obesity [34–37]. The current study of youth and adults showed that the prevalence of being overweight or obesity was 16.9 %. In contrast to other countries, the prevalence of overweight or obesity among patients with T1D in China was low. Another study[38] from China showed that in Chinese children and adolescents, mean BMI and the prevalence of overweight or obesity steadily increased from 19,991 to 2006. However, the most noticeable increase was in children from urban areas and those from higher income backgrounds, indicating a strong correlation between BMI and quality of life. In this study, the prevalence of overweight or obesity in T1D patients has not shown an upward trend in recent years (Fig. 2), which may suggest that there is still much room for improvement in the quality of care for T1D patients in China.

Dyslipidemia is a common concomitant disease of T1D, which is very related to glucose control. The overall prevalence of dyslipidemia among patients with T1D in this study was 37.8 %, which was higher than that found in Germany at 28.6 %[39] and lower than that in Korean at 37.9 %[40]. In contrast, the prevalence in the present study was much lower than that found in Bangladesh, which reported the overall prevalence of dyslipidemia were 65 % in youth with T1D [41]. Also, a higher prevalence was found in Brazilian which reported that the prevalence of dyslipidemia in young patients was72.5 %[42]. The wide range of prevalence in various studies may be due to differences of treatment level.

Since this study was a retrospective analysis, data was limited about protein tyrosine phosphatase 2 auto- antibody (IA-2 A) and zinc transporter-8 autoantibody (ZnT8A). Therefore, it is a pity that we only analyzed the proportion of GADA, ICA, IAA. We observed that the prevalence of GADA, ICA, IAA in the present study were 28.1 %, 22.8% and 2.6 %, respectively, which were extremely lower than results reported in other countries [43–45]. A study from China by Xiao et al. showed that the positive rate of GADA was 52.2 %, IAA was 23.9 %, and ICA was 14.3 %. Another study[46] from China showed that the prevalence of GADA was 56.3 % at diagnosis, decreasing to 50.5 % one year later, and 43.3 % 3–5 years later. The reported prevalence of these autoantibodies in T1D may vary greatly depending on disease duration, age of onset, antibody assay method and so on. One possibility for the low prevalence of islet-related antibodies in this study is that other autoantibodies yet to be included maybe contributory. Another possibility is the low genetic susceptibilities in this population. Diabetes-related autoantibodies appeared in people with an increased genetic risk of T1D[47]. A study showed that the frequency of GADA is only 30–40 % in Asian T1DM patients[48]. It attributed this difference to the fact that autoimmune-mediated β -cell destruction is less common in Asian patients. Besides, the older age of patients in this study maybe associated with the low prevalence of antibodies. A study demonstrated that with the increase of the age of onset, the newly diagnosed patients without any islet autoantibodies also increased[49]. Besides, compared with patients over 15 years old at onset, IAA was more common in children under 5 years old who developed T1D[49].

### Comparison of clinical features in young-onset and adult-onset patients

It was generally accepted that TID in children were considered to have acute onset with classical symptoms of diabetes, while in adults, the onset of T1D can be more variable and have a better residual beta-cell function. In the analysis of 453 newly diagnosed patients, we observed that adult-onset patients accounted for 48.3 %. Adult-onset patients comprised better islet function reflected by FCP and 2-h CP. This result was in line with the results of the Diabetes Control and Complications Trial study and other subsequent studies[50–52]. It should be noted that the median fasting C-peptide level in young-onset group was 0.3ng/ml, which was lower than a report from the USA (median random serum C-peptide of children was 0.4ng/ml). Besides, the current study found that patients with the adult-onset group had lower median HbA1c levels and incidence of DKA at disease onset, which indicated that residual β-cell function was better, clinical manifestation was less severe, or hyperglycemia lasted shorter before diagnosis. Different from the traditional view that autoimmune disease are mostly women, T1D is even characterized by a clear male predominance in diabetes for new cases in Caucasians[53]. Similar to previous findings, we found the male excess in present population. In addition, adult-onset group had higher proportion of male patients than young-onset group, agreeing with other studies in China[23, 24]. As previously reported, studies had shown that the adult patients were most frequently tested positive for GADA[54], which indicated that very young patients tend to mount a weak antibody response to GADA and that the intensity of the GADA response increases with age[55].

The limitation of this study is the fact that this study is a retrospective and hospital-based study from one center in Henan province, so the representativeness of the research results is not as good as that of multi-centers. Besides, due to the limitations of clinical diagnosis, some younger patients could have a monogenic diabetes, or the older subjects could have maturity-onset diabetes of youth (MODY). It is possible that some patients diagnosed as T1D may actually have T2D if studied further. Last but not least, as the value of the variables of interest were not measured or recorded for all subjects in the analysis, there were lots of missing data, especially the data of BMI and islet-related antibodies. Although available-case analysis and multiple imputation were adopted to address the presence of missing data, these approaches can lead to biased estimates of statistics.

## Conclusions

In conclusion, despite the limitations of research design and data analysis, our data indicated that patients with T1D in this area were characterized by poor overall blood glucose control, high prevalence of diabetic ketoacidosis, dyslipidemia and diabetic retinopathy, and low prevalence of islet-related antibodies, and overweight or obesity. Compared with young-onset patients, adult-onset patients with T1D were not uncommon and had better clinical manifestations at disease onset than young-onset patients. This may have some guiding effects on the clinic. Any findings related to BMI and autoantibodies should be considered strictly exploratory due to excessive missing data. 

## Data Availability

The data that support the findings of this study are available from the authors and will be shared upon reasonable request.

## Abbreviations

GADA: Glutamic acid decarboxylase
ICA: Islet-cell antibodies
IAA: Insulin antibodies
TC: Total cholesterol
TG: Triglyceride
HDL-C: High-density lipoprotein cholesterol
LDL-C: Low-density lipoprotein cholesterol
FCP: Fasting serum C-peptide
2-h CP: 2-h postprandial serum C-peptide
